# Prostate cancer outcomes in France: treatments, adverse effects and two-year mortality

**DOI:** 10.1186/1471-2490-14-48

**Published:** 2014-06-13

**Authors:** Philippe Tuppin, Solène Samson, Anne Fagot-Campagna, Bertrand Lukacs, François Alla, Fred Paccaud, Jean-Christophe Thalabard, Eric Vicaut, Michel Vidaud, Bertrand Millat

**Affiliations:** 1 National general health insurance scheme (Caisse Nationale d’Assurance Maladie des Travailleurs Salariés, CNAMTS), Paris, France; 2 Department of Urology, Tenon Hospital, Assistance Publique- Hôpitaux de Paris, 4 Rue de la Chine, 75020 Paris, France

**Keywords:** Prostate cancer, Treatments, Adverse effects, Prostatectomy, Radiotherapy, Complications, Population-based study

## Abstract

**Background:**

This very large population-based study investigated outcomes after a diagnosis of prostate cancer (PCa) in terms of mortality rates, treatments and adverse effects.

**Methods:**

Among the 11 million men aged 40 years and over covered by the general national health insurance scheme, those with newly managed PCa in 2009 were followed for two years based on data from the national health insurance information system (SNIIRAM). Patients were identified using hospitalisation diagnoses and specific refunds related to PCa and PCa treatments. Adverse effects of PCa treatments were identified by using hospital diagnoses, specific procedures and drug refunds.

**Results:**

The age-standardised two-year all-cause mortality rate among the 43,460 men included in the study was 8.4%, twice that of all men aged 40 years and over. Among the 36,734 two-year survivors, 38% had undergone prostatectomy, 36% had been treated by hormone therapy, 29% by radiotherapy, 3% by brachytherapy and 20% were not treated. The frequency of treatment-related adverse effects varied according to age and type of treatment. Among men between 50 and 69 years of age treated by prostatectomy alone, 61% were treated for erectile dysfunction and 24% were treated for urinary disorders. The frequency of treatment for these disorders decreased during the second year compared to the first year (erectile dysfunction: 41% vs 53%, urinary disorders: 9% vs 20%). The frequencies of these treatments among men treated by external beam radiotherapy alone were 7% and 14%, respectively. Among men between 50 and 69 years with treated PCa, 46% received treatments for erectile dysfunction and 22% for urinary disorders. For controls without PCa but treated surgically for benign prostatic hyperplasia, these frequencies were 1.5% and 6.0%, respectively.

**Conclusions:**

We report high survival rates two years after a diagnosis of PCa, but a high frequency of PCa treatment-related adverse effects. These frequencies remain underestimated, as they are based on treatments for erectile dysfunction and urinary disorders and do not reflect all functional outcomes. These results should help urologists and general practitioners to inform their patients about outcomes at the time of screening and diagnosis, and especially about potential treatment-related adverse effects.

## Background

The availability of serum prostate-specific antigen (PSA) testing has led to a dramatic increase in the incidence of localised prostate cancer (PCa), corresponding to well or moderately differentiated tumours with a low risk of progression [1-4]. The discordant results of randomised studies concerning the impact of PSA testing on mortality and the adverse effects of treatments contribute to the debate on overdiagnosis and overtreatment of PCa [4-13].

Adverse effects on sexual and urinary functions are due to specific pathophysiological mechanisms related to each of the main treatment modalities, e.g., radical prostatectomy, brachytherapy, external beam radiotherapy and hormone therapy. Patient quality of life is usually estimated by assessment tools filled in by the patient or completed during telephone interviews, based on samples of less than 2000 individuals [7-13]. The frequency of symptoms is generally high, but depends on the definition used for urinary and erectile problems, as well as on practices, measurement tools and monitoring periods. Quality of life assessment rarely includes comparisons with a control group without PCa.

The purpose of this observational study based on a large population of French men aged 40 and over with newly managed PCa in 2009 was to estimate the two-year all-cause mortality according to the various treatments for PCa, and the frequency of PCa treatments and their adverse effects, when they required health care resources, for survivors compared to two control groups.

## Methods

### Population and data source

All data were obtained from the French National Health Insurance Information System (SNIIRAM) [14]. Access to data and data analysis are granted by decree only for a period of three years plus the current year and authorisation from the French Data protection Authority (*Commission nationale de l'informatique et des libertés, CNIL*) must also be obtained. This database comprehensively records all hospitalisations, prescriptions, health care services and procedures reimbursed together with their dates. Drugs are identified using their ATC (Anatomical Therapeutic Classification) code, while medical and surgical procedures are identified by a specific classification. The SNIIRAM does not contain any medical information concerning results related to prescriptions or examinations. It nevertheless includes information on the presence of long-term chronic diseases (LTCD), such as PCa, eligible for 100% reimbursement of health care expenditure, after approval by a national health insurance consultant physician. LTCD status is granted at the time of diagnosis of the disease and is renewable for five years. These chronic diseases and hospital diagnoses are coded according to the International Classification of Diseases (ICD 10). The SNIIRAM also includes a permanent sample (EGB: échantillon généraliste des bénéficiares) of individuals selected at random from 1/97^th^ of all beneficiaries of the French general health insurance schemes, i.e. approximately 600,000 individuals.

The study population was composed of men aged 40 years and over, covered by the general health insurance scheme, with newly managed PCa in 2009. In 2009, 73% of French men aged 40 and over (11 million people) were covered by the general health insurance scheme. The rest of the French population was covered by other specific health insurance schemes. One of the reasons for limiting the study to individuals covered by the general scheme was the availability of survival status and date of death from the National Institute for Statistics and Economic Studies for the study period.

### Definitions and data analysis

Men with PCa were identified by the presence of at least one of the following criteria in 2009: a hospital stay with a PCa code as primary diagnosis, PCa *in situ* or prostate tumour with an unpredictable course; LTCD for PCa; hospitalisation for radical prostatectomy, subcapsular orchidectomy or specific brachytherapy; hospitalisation for chemotherapy or radiotherapy with a PCa-related diagnosis; at least one reimbursement for GnRH analogues, antiandrogens or oestrogens and estramustine. To select only newly diagnosed cases in 2009, men with any of the above criteria in 2008 were excluded.

PCa treatments and their combinations were identified during the two-year follow-up after the first treatment criterion identified in 2009. Two groups of patients without treatment over the next two years were formed: one group with a hospitalisation corresponding to a primary diagnosis of PCa (with or without LTCD for PCa in 2009) and the other group with only LTCD status for PCa granted in 2009. These patients were considered to be on watchful waiting for PCa. For the study of treatment combinations, subcapsular orchidectomy, which is rarely performed, was grouped together with hormone therapy.

Complications were defined on the basis of at least one hospitalisation with a main diagnosis corresponding to the specific codes of complications or the presence of a surgical procedure to treat these complications. Urinary disorders were identified by reimbursements of external devices to treat incontinence (urine collectors and penile sheaths). Reimbursements of specific drugs were also considered (ATC G04BD: oxybutynin, flavoxate, trospium and solifenacin). Erectile dysfunction was identified by reimbursements for penile prostheses or intracavernous injections of vasoactive agents (sildenafil was not reimbursed). Drug treatments were defined on the basis of at least one reimbursement for specific drugs for patients still alive at one year and at two years.

The two-year mortality rate for patients with treated PCa was compared to that of all male health insurance beneficiaries aged 40 years and over. A standardised mortality ratio (SMR) was calculated for each age group and the overall SMR was age-standardised using data for men aged 40 years and over in the general scheme. Frequencies of treatments and complications were studied among men of the general scheme who were still alive with a follow-up of two years. Among men between the ages of 50 and 69, complication rates according to the type of medical treatment were compared to those of two control groups without PCa, selected from the EGB according to the presence of a surgical or endoscopic procedure performed for BPH between 2008 and 2011. The 50–69 years age-group was selected because radical prostatectomy, associated with a higher complication rate than other treatments, is more frequent at these ages and also has a greater impact on erectile dysfunction and quality of life and is therefore more frequently treated. The frequencies for each of these groups were age-standardised for all males aged 50–69. Chi-square tests were used to compare frequencies between groups. Statistical analyses were performed using SAS Enterprise Guide 4.3 software (SAS Institute Inc., Cary, NC, USA).

## Results

### Survival

Newly diagnosed PCa in 2009 was found in 43,460 men with a mean age of 70.2 years. The crude one-year and two-year all-cause mortality rates were 7% and 13%, respectively, and the age-standardised rates were 4.6% and 8.4%, respectively (Table 1). The age-standardised mortality rate was slightly higher before the age of 50 (7%) and was 47% for men aged 85 years and over. The patients of this population had a significant excess two-year all-cause mortality rate compared to all men aged 40 and over (SMR = 2). The SMR was higher for the youngest age groups. In this group of 43,460 patients, 1,169 subjects still alive at two years were excluded because they were no longer covered by the general health insurance scheme or had been admitted to an institution that managed medicinal products directly with no reimbursements reported in the SNIIRAM database.

**Table 1:** One-year and two-year mortality rates among men with newly managed prostate cancer in 2009 by age and comparison with all male general scheme beneficiaries aged 40 and over, according to age after two years

	**40-49**	**50-54**	**55-59**	**60-64**	**65-69**	**70-74**	**75-79**	**80-84**	**85 and over**	**All ages**
**Patients with cancer**										
**N**	**478**	**1,567**	**4,079**	**7,371**	**7,435**	**7,876**	**6,663**	**4,351**	**3,640**	**43,460**
%	1.1	3.6	9.4	17.0	17.1	18.1	15.3	10.0	8.4	100.0
**One-year follow-up**										
Patients still alive*	443	1,481	3,857	7,026	7,070	7,368	6,022	3,568	2,526	39,361
Deceased										
N	15	40	101	186	242	392	532	727	1,047	3,282
%	3.3	2.6	2.6	2.6	3.3	5.1	8.1	16.9	29.3	4.6**
**Two-year follow-up**										
Patients still alive*	414	1,433	3,727	6,782	6,792	7,033	5,554	3,106	1,893	36,734
Deceased										
N	31	71	192	369	456	665	948	1,159	1,656	5,547
%	7.0	4.7	4.9	5.2	6.3	8.6	14.6	27.2	46.7	8.4**
SMR^§^	11.4	3.7	2.6	2.1	1.9	1.8	1.9	2.1	1.9	2.0**

*Excluding deaths, regime changes and patients lost to follow-up.

**Age-standardised.

^§^p < 0.001 for all SMR.

Age-standardised two-year mortality rates varied according to age and type of PCa treatment (Table 2). Lowest two-year mortality rates were observed for patients treated by radical prostatectomy (4%) and hormone therapy (9%) and highest two-year mortality rates were observed for patients treated by external beam radiotherapy (14% overall, and 30% for those treated by external beam radiotherapy alone) and chemotherapy (38%). The two-year mortality rate for men not treated for PCa was 15% for men hospitalised for PCa and 8% for men with only LTCD status.

**Table 2:** Age-standardised two-year mortality rates among men with newly managed prostate cancer (PCa) in 2009 according to treatment, and frequency of treatments and their combinations among men still alive at two-years according to age

	**Death rate**	**Frequency of treatment**									
	**Age-standardised**	**40-49**	**50-54**	**55-59**	**60-64**	**65-69**	**70-74**	**75-79**	**80-84**	**≥85**	**Total**
**N**	**43,460**	**414**	**1,433**	**3,727**	**6,782**	**6,792**	**7,033**	**5,554**	**3,106**	**1,893**	**36,734**
**Treatments (at least one)**	%	%	%	%	%	%	%	%	%	%	%
Total prostatectomy	4.3	46.9	66.9	65.8	60.6	54.2	32.4	6.0	0.2	0.3	38.1
- laparoscopy	3.5	20.3	37.7	36.6	33.2	30.9	19.7	3.8	0.1	0.2	21.6
- laparotomy	5.1	26.6	29.2	29.2	27.4	23.3	12.7	2.1	0.1	0.1	16.5
Hormone therapy	9.4	40.3	21.4	20.3	20.5	25.4	34.9	53.3	65.4	66.8	35.5
Subcapsular orchidectomy	12.7	1.2	0.3	0.2	0.1	0.1	0.2	0.4	1.2	1.3	0.3
Chemotherapy	38.5	4.3	2.5	1.6	1.6	1.4	1.5	1.7	1.8	1.1	1.6
Brachytherapy	0.8	2.4	3.0	4.2	4.0	2.8	2.7	1.2	0.1	0.0	2.5
External beam radiotherapy	14.0	18.4	21.1	24.8	26.0	28.8	39.7	40.6	12.2	3.5	28.6
No treatment*:	13.6	12.1	13.9	14.7	16.9	17.8	20.7	24.6	30.2	31.2	20.4
- Hospitalisation with PCa diagnosis	15.0	10.4	11.0	11.5	12.8	13.6	15.3	17.9	22.9	25.5	15.5
- Isolated LTCD for PCa	8.1	1.7	2.9	3.2	4.2	4.2	5.3	6.7	7.2	5.7	4.9
**Treatment combinations**	%	%	%	%	%	%	%	%	%	%	%
**Prostatectomy, all types**											
Alone	4.4	37.4	53.5	52.3	49.4	43.7	25.4	4.8	0.2	0.1	30.6
Combined with:											
- Hormone therapy	1.2	1.9	3.6	3.4	2.8	3.0	2.1	0.6	0.0	0.1	2.1
- External beam radiotherapy	4.2	4.1	5.7	5.6	5.0	4.1	2.8	0.4	0.0	0.0	3.1
- External beam radiotherapy	0.4	2.9	4.0	4.1	3.2	3.2	1.9	0.3	0.0	0.0	2.2
+ Hormone therapy											
**Hormone therapy**											
Alone	9.5	28.3	6.8	4.0	3.8	5.6	9.8	26.4	55.9	64.0	16.6
Combined with:											
- External beam radiotherapy	8.9	4.3	4.9	6.7	8.7	11.6	19.1	23.9	8.5	2.5	12.8
**External beam radiotherapy alone**	30.0	2.2	2.5	3.4	4.7	6.7	12.9	14.3	3.0	0.7	7.5

LTCD: long term chronic disease status.

*Absence of the above treatments during the two-year follow-up.

### Prostate cancer treatment

In the population of 36,734 men with PCa still alive after two years, 38% had been treated by radical prostatectomy, 35% by hormone therapy, 29% by external beam radiotherapy, 2.5% by brachytherapy, 0.3% by subcapsular orchidectomy and 1.6% by chemotherapy (2). Twenty per cent of patients had not received any active treatment, but had been hospitalised with a specific code for PCa (15%) or had been granted LTCD status (5%).

The frequency of prostatectomy decreased from 67% in men between the ages of 50 and 54 to 6% among men between the ages of 75 and 79. The frequency of hormone therapy, stable at around 20% between the ages of 50 and 64, increased to 65% for men aged 80 and over. The frequency of external beam radiotherapy reached a peak of 41% in men between the ages of 75 and 79. The most common treatment combination was hormone therapy and external beam radiotherapy (13%). Prostatectomy was often performed alone (31%), especially among the youngest patients (more than half), and was combined with radiotherapy and hormone therapy in only 2% of men treated for PCa.

### Adverse effects of PCa treatments

In the population of 36,734 men with PCa still alive after two years, regardless of the PCa treatment, 54% presented no health care consumption corresponding to adverse effects of PCa treatments (Table 3).

**Table 3:** Frequency of treatments* for various complications, according to age, among men with newly managed prostate cancer in 2009, still alive after two years

	**40-49**	**50-54**	**55-59**	**60-64**	**65-69**	**70-74**	**75-79**	**80-84**	**≥85**	**All ages**
**Complications**	**414**	**1,433**	**3,727**	**6,782**	**6,792**	**7,033**	**5,554**	**3,106**	**1,893**	**36,734**
	**%**	**%**	**%**	**%**	**%**	**%**	**%**	**%**	**%**	**%**
**Erectile dysfunction**	34.1	46.9	43.9	37.1	27.6	11.8	2.1	0.5	0.1	21.2
Intracavernous injection	33.8	46.7	43.8	37.0	27.5	11.7	2.0	0.4	0.1	21.1
Penile prosthesis	0.2	0.2	0.2	0.1	0.1	0.1	0.0	0.0	0.0	0.1
**Urinary disorders**	14.0	20.4	20.5	19.4	21.8	19.5	17.1	14.6	14.4	18.9
Drug treatment	10.6	15.6	16.4	14.7	17.1	15.9	14.5	11.6	9.3	15.0
External devices	3.9	6.1	5.6	5.8	6.2	4.8	2.6	2.8	4.2	4.8
Surgical procedure	1.0	1.0	1.3	1.4	1.6	1.2	0.4	0.1	0.0	1.0
**Clot removal**										
Surgical procedure	1.2	1.4	1.0	1.3	1.0	1.3	1.4	1.9	2.3	1.4
**Urethral stricture**										
Surgical procedure	1.4	1.5	1.4	1.5	2.0	2.4	2.0	2.0	2.6	1.9
**Bladder neck stenosis**										
Surgical procedure	1.4	2.2	1.7	1.8	1.9	2.0	1.8	1.3	1.2	1.8
**Radiation-induced inflammation**										
Hospitalisation	0.0	0.6	0.5	0.6	0.9	1.4	2.0	0.5	0.1	1.0
**Acute urinary retention**										
Surgical procedure	5.1	3.8	5.4	7.5	8.6	12.6	17.2	20.6	19.4	11.5
**Urethral or rectal fistula**										
Hospitalisation	0.7	0.4	0.2	0.2	0.3	0.1	0.1	0.0	0.0	0.2
**None**	55.1	40.5	40.5	45.2	49.4	60.0	65.4	66.4	64.3	54.1
**Drug treatment**										
Antidepressants	14.5	15.6	12.4	9.2	9.4	9.3	11.6	15.9	18.8	11.3
Anxiolytics	19.3	19.0	17.2	15.0	15.4	17.0	18.2	21.7	23.9	17.4

*Hospitalisation: primary diagnosis, surgical treatment: presence of a specific surgical procedure code, external devices and medication: reimbursement codes.

The frequency of reimbursement for at least one intracavernous injection for erectile dysfunction was 21% for all ages and ranged between 33% and 47% for men under the age of 65. A penile prosthesis was used by 0.2% of men under 55 years of age. The frequency of reimbursement for drugs used to treat urinary disorders was 15% for all ages, and varied slightly according to age (from 9% to 17%), while external devices were reimbursed in 4.8% of cases and 1% of cases were treated by surgery. The frequency of surgical procedures for acute urinary retention was 11% for all ages, but was higher among older men. Surgical treatment of complications was more common during the first year of follow-up.

The frequency of treatment for adverse effects varied according to the type of PCa treatment (Table 4). Among patients between the ages of 50 and 69, intracavernous injection for erectile dysfunction was more common for patients treated by prostatectomy alone (61%) compared to patients treated by radiotherapy alone (7%). Similar results were observed for urinary disorders, with reimbursement of drugs in 18% versus 12% of patients and reimbursement of external devices in 8% and 2% of patients, respectively. Patients treated by prostatectomy alone more frequently received reimbursements for drugs used to treat erectile dysfunction than those treated by prostatectomy combined with external beam radiotherapy or hormone therapy.

**Table 4:** Frequencies of the various complications according to type of treatment* among men aged between 50 and 69 with newly managed prostate cancer (PCa) in 2009 and still alive after two years, and comparison with two control groups without PCa, with or without surgical treatment for benign prostatic hyperplasia (BPH)

	**Prostatectomy**		**External beam radiotherapy**	**Hormone therapy**	**Treated**	**Untreated**	**Control groups without PCA**	
					**PCa**	PCa***		
	**Alone**	With another treatment**	**Alone**	**Alone**	**All**		**Without BPH surgery**	**With BPH surgery**
**N**	**9,035**	**2,159**	**941**	**882**	**15,623**	**3,107**	**43,099**	**1,026**
**Mean age (years)**	**61.8**	**61.8**	**63.6**	**62.4**	**62.1**	**62.5**	**57.8**	**60.5**
**Complications**	**%**	**%**	**%**		**%**	**%**	**%**	**%**
**Erectile dysfunction**								
Two years	61.4	47.9°	7.2°	6.8°	45.8	9.6°	0.3°	1.5°
Drug treatment	61.3	47.9	7.2	6.8	45.7	9.5	0.3	1.5
Penile prosthesis	0.2	0.2	0.04	0.0	0.2	0.1	0.00	0.00
First year	**53.2**	**38.1°**	**2.6°**	**4.7°**	**38.7**	**8.1°**	**0.2°**	**1.0°**
Drug treatment	53.1	38.1	2.6	4.7	38.6	8.0	0.2	1.0
Penile prosthesis	0.1	0.0	0.0	0.0	0.0	0.0	0.0	0.0
Second year	**41.5**	**31.8°**	**6.3°**	**4.4°**	**30.8**	**6.4°**	**0.2°**	**1.1°**
Drug treatment	41.3	31.8	6.3	4.4	30.7	6.3	0.2	1.1
Penile prosthesis	0.2	0.2	0.0	0.0	0.1	0.0	0.0	0.0
**Urinary disorders**								
Two years	**23.7**	**27.9°**	**14.4°**	**8.7°**	**21.6**	**12.3°**	**0.4°**	**5.6°**
Drug treatment	18.0	21.6	12.3	7.4	17.0	9.4	0.3	4.8
External devices	7.7	9.3	2.4	1.8	6.3	3.8	0.1	0.8
Surgical procedure	2.1	1.2	0.00	0.1	1.4	0.6	0.00	0.00
First year	**20.1**	**22.8**	**10.9°**	**4.7°**	**17.5**	**10.4°**	**0.2°**	**3.7°**
Drug treatment	14.7	16.0	8.9	4.0	13.1	7.5	0.2	3.2
External devices	7.2	8.9	2.2	1.1	5.8	3.3	0.1	0.5
Surgical procedure	0.5	0.2	0.0	0.1	0.3	0.5	0.0	0.0
Second year	9.0	11.5°	7.6	6.0°	9.0	5.5°	0.2°	3.5°
Drug treatment	7.2	10.3	5.7	4.8	7.6	4.2	0.2	3.0
External devices	1.4	1.5	1.9	1.2	1.3	1.6	0.1	0.4
Surgical procedure	1.7	1.0	0.0	0.1	1.1	0.2	0.0	0.0
**Clot removal**								
Surgical procedure	1.2	1.2	1.0	1.3	1.2	1.2	0.00°	0.3°
**Urethral stricture**								
Surgical procedure	1.6	1.9	1.5	1.1	1.4	2.2	0.0°	1.3
**Bladder neck stenosis**								
Surgical procedure	2.0	2.2	0.3°	1.0°	1.9	1.7	0.00°	0.9°
**Radiation-induced inflammation**								
Hospitalisation	0.0	1.1°	3.2°	0.1	0.7	0.3°	0.00°	0.00°
**Acute urinary retention**								
Surgical procedure	1.4	1.3	12.2	9.9°	3.5	19.3°	0.00°	3.6
**Urethral-rectal fistula**								
Hospitalisation	0.5	0.4	0.00	0.1°	0.3	0.0°	0.00°	0.00°
**None**	28.1	35.8°	69.3°	76.9°	40.1	63.5°	99.3°	89.0°
**Drug treatment**								
Antidepressants	11.3	11.4	12.2	15.9	12.0	12.2	7.1°	11.7
Anxiolytics	15.3	18.1	21.1°	19.8	17.1	15.0°	10.4°	16.5

*Hospitalisation: primary diagnosis, surgical treatment: presence of a specific surgical procedure code, external devices and medication: reimbursement codes.

**Patients without treatment during the two years but with a primary diagnosis of PCa during hospitalisation or long-term chronic disease status for PCa.

***External beam radiotherapy and/or hormone therapy.

Frequencies are age-standardised for men aged between 50 and 69 covered by the general scheme.

°p < 0.05 for comparisons of frequencies between prostatectomy alone and prostatectomy combined with another treatment, external beam radiotherapy, for comparisons between untreated PCa and treated PCa, for comparisons between treated PCa and each control group.

Among patients with untreated PCa, 10% had received medication for erectile dysfunction, 12% had received medication for urinary disorders, and 19% had received medication for acute urinary retention. For the subgroup of patients not hospitalised for PCa, these frequencies were 20%, 12% and 5%, respectively. In contrast, the frequencies of the various treatments for erectile dysfunction and urinary disorders in the two control groups without PCa were very low, even in men who had undergone BPH surgery. The highest treatment frequencies observed in these men concerned medication for urinary disorders (4.8%), acute urinary retention (3.6%) and, to a lesser degree, erectile dysfunction (1.5%).

The frequency of treatment for erectile dysfunction decreased during the second year compared to the first year among patients treated by radical prostatectomy and patients with untreated PCa, while the frequency of treatment for erectile dysfunction increased between the first and second year among men treated by external beam radiotherapy alone. The frequency of drug treatment for urinary disorders decreased during the second year for men treated by prostatectomy and men with untreated PCa. However, men with untreated PCa had a high frequency of treatment for acute urinary retention.

## Discussion

This study reports outcomes for men diagnosed with PCa based on health care consumption data concerning a very large population followed for two years. We report a low 8% mortality rate which is nevertheless twofold higher than that of the general male population and a higher frequency of radical treatments in men under the age of 70. Very high frequencies of erectile dysfunction and urinary disorders requiring treatment were observed among men between the ages of 50 and 69 treated by radical prostatectomy, compared to controls and men with untreated PCa.

This study demonstrated a crude one-year survival rate of 95% with variations related to age, which is similar to estimates from French cancer registries (92% one-year overall survival, and a specific survival rate of 96%) [15]. Causes of death other than PCa were reported for 80% of deceased patients in the United States and 65% of deceased patients in Sweden [16]. Patients with PCa, especially with metastatic disease, presented a higher risk of death by suicide or cardiovascular disease than the general population [17]. These data highlight the high survival rate and the fact that mortality is due to causes other than PCa. Although no information was available concerning specific mortality rates, the two-year mortality rate in this population was twofold higher than that of men covered by the general health insurance scheme. More specifically, the highest SMR was observed for the 40–49 year age-group, which was less frequently treated by prostatectomy and more frequently treated by chemotherapy and hormone therapy, which could suggest more advanced disease, although no information on disease stage was available in our database.

In this study, the estimated frequencies of the various treatments for PCa were 38% for prostatectomy, 35% for hormone therapy, 29% for external beam radiotherapy and 20% of patients did not receive any treatment during the two-year follow-up. This group can be considered to correspond to watchful waiting, but some of these patients may have received treatment for PCa before 2008 and were wrongly considered to have newly diagnosed PCa. However, it is much more likely that the watchful waiting group was underestimated, as newly diagnosed patients who were not hospitalised and who did not require treatment or LTCD status were not included in this database. A French registry study conducted in 2001 on 1,840 men of all ages with stage T1 or T2 PCa reported slightly lower frequencies for prostatectomy (31%) and hormone therapy (22%) and nearly the same rate for radiotherapy (26%) and watchful waiting (19%) [18]. The proportion of patients managed by watchful waiting has most likely increased since 2001. The more recent Swedish registry provides different figures. Among the 31,903 patients of all ages with localised PCa between 1996 and 2006, 41% had undergone prostatectomy (66% between 55 and 59 years of age, as for all stages in this study), 20% had received external beam radiotherapy (24% for those between 65 and 69) and 30%, a very high proportion, were on watchful waiting [19]. The Australian state of New South Wales registry showed that, in a group of 1,636 patients under the age of 70 with localised PCa between 2000 and 2002, 60% had been treated by prostatectomy, 28% by external beam radiotherapy, 14% by hormone therapy and 12% by watchful waiting [8]. For the same age-group, at all stages, our study found similar frequencies for prostatectomy (60%) and external beam radiotherapy (26%) but higher frequencies for hormone therapy (23%) and watchful waiting (16%). The choice of treatment probably varies from one series to another depending on the tumour grades included, the study period, patient characteristics and comorbidity. However, it is very likely that the choice of treatment also depends on PCa screening frequency and medical practices. For example, brachytherapy is less commonly used in France [20].

This study demonstrated a very high rate of PCa treatment-related adverse effects. Over a 2-year period, treatments for urinary and erectile disorders were reported in 19% and 21% of all men with newly diagnosed Pca, respectively. These rates were even higher among men aged 50–54 years (20% and 47%) and men of all ages who had undergone prostatectomy (24% and 61%). In contrast with studies on urinary disorders and erectile dysfunction based on the presence of symptoms or quality of life assessment, using various definitions and instruments, in the present study, these disorders were estimated on the basis of health care consumption. These estimates depend on the patient’s perception of the disorder, the efficacy of treatment of the disorder and the physician’s or surgeon’s impression.

These adverse effect rates, although underestimated, can nevertheless be considered to accurately reflect the impact of PCa treatments on quality of life. Specific health care consumption was very rare in the control group without PCa and who had not undergone BPH surgery. As expected, treatment rates for acute urinary retention and urinary disorders were higher among controls that had undergone BPH surgery. Higher rates of treatment for acute urinary retention were observed for men with untreated PCa compared to men with treated PCa, which may be linked to tumour progression [13]. As also reported in quality of life studies, lower rates of adverse effects were observed after external beam radiotherapy than after prostatectomy [7]–[13]. In our study, surgery for incontinence during the two years following prostatectomy was performed in 2.1% of patients versus 2.6% in a Canadian study based on 25,436 patients of all ages with a five-year follow-up [21,22]. Patients treated by prostatectomy in combination with another treatment such as hormone therapy also had a lower rate of treatment for erectile disorders, which could be explained by the lack of available treatment for sexual dysfunction induced by hormone therapy.

Urinary disorders and erectile dysfunction are much less common after external beam radiotherapy: 7% of patients experienced erectile dysfunction and 15% experienced urinary disorders. A recent American study based on 1,201 patients of all ages with stage T1 or T2 PCa between 2003 and 2006, analysing first-line treatments, reported urinary disorders in 30% of patients after two months and 7% of patients after two years [10]. The present study showed lower rates of adverse effects of treatment after the first year following PCa treatment, in line with the results of quality of life studies that reported improvement of symptoms after the first year of treatment, with an impact on quality of life, but without return to baseline conditions [7,8,10,11].

This study presents a number of limitations. Certain groups of the French population (25%) were not included due to their occupation, such as civil servants, liberal professions and farmers, which may influence epidemiological characteristics (exposure) or health care consumption, and who are covered by other health insurance schemes. The data of this study were derived from administrative databases comprising the classical limitations concerning the modalities of data collection and coding. However, these databases are useful for economic purposes for hospitals and ambulatory services, which could be an argument in favour of the quality and exhaustiveness of data on the medicinal products and health care provided. The availability of data for a period of three years plus the current year constitutes a limit to the duration of follow-up of prospective studies, especially concerning medium-term or long-term adverse effects, such as those of hormone therapy. Some products, such as sildenafil or vacuum pumps, are not reimbursed. The database does not include any information on PCa stage, type of tumour or the severity of the disorders, in contrast with qualitative studies, and only allows analysis of treatments prescribed and reimbursed. PCa considered to be newly managed could also correspond to recurrences requiring further treatment, but a one- to two-year treatment-free period prior to inclusion was required. These patients were also required to have LTCD status for PCa granted before 2009. As already emphasized, outcomes based on health care consumption data may not reflect all complications experienced nor their severity, but this aspect must be considered case by case for each database [23,24]. The strengths of our observational study are that it was based on a very large, comprehensive population of men covered by the French national health insurance scheme and comprised comparisons with control groups. Given the differences in health care systems between Western countries, the results of this study cannot be directly extrapolated in other settings. In France, PCa incidence was estimated to be 71,000 cases for 2011 using data from regional cancer registries. Recently, a new estimation provided a lower incidence of 53,465 cases for 2009. National extrapolation of the 43,460 cases of PCa in 2009 identified by this study would give an incidence of 59,500 cases, close to the 2009 estimation.

## Conclusions

In this population of men with treated or diagnosed PCa, we report, as expected, a high two-year survival rate and describe the frequencies of various PCa treatments. More importantly, we report a high frequency of men treated for adverse effects induced by PCa treatments, especially prostatectomy. These results can be used as a basis to provide patients with information about outcomes at the time of screening and diagnosis, and to choose treatments according to national guidelines and PCa clinical characteristics.

## Abbreviations

PSA: Prostate-specific antigen; PCa: Prostate cancer; SNIIRAM: National health insurance information system (SNIIRAM); LTCD: Long-term chronic diseases; INSEE: National institute for statistics and economic studies; ICD10: International classification of diseases 10 revision; ATC: Anatomical therapeutic classification.

## Competing interests

All authors declare that they have no competing financial interests. There was no specific funding for this study, which was conducted by the employees of the French national health insurance general scheme.

## Authors’ contributions

PT participated in the conception and design, data interpretation, manuscript drafting, SS participated in design, data analysis and interpretation. AFC, BL and FA participated in design and data interpretation. FP, JCT, EV, MV and BM participated in design, interpretation, manuscript validation and supervision. All authors have read and approved the final manuscript.

## Pre-publication history

The pre-publication history for this paper can be accessed here:

http://www.biomedcentral.com/1471-2490/14/48/prepub
